# An Overview of Upland Peatlands’ Vegetation of Apennines, Sicily, and Sardinia (Italy)

**DOI:** 10.3390/plants14131931

**Published:** 2025-06-23

**Authors:** Enrico Bajona, Emilio Di Gristina, Giuseppe Venturella

**Affiliations:** 1 Department of Earth and Marine Sciences (DiSTeM), University of Palermo, Via Archirafi 20, 90123 Palermo, Italy; enrico.bajona@unipa.it; 2 National Biodiversity Future Centre (NBFC), Palermo Piazza Marina 61, 90133 Palermo, Italy; giuseppe.venturella@unipa.it; 3 Department of Agricultural, Food and Forest Sciences (SAAF), University of Palermo, Viale delle Scienze, Ed. 4, 90128 Palermo, Italy

**Keywords:** Mediterranean vegetation, wetlands, mountain mires, vegetation monitoring, vegetation resurvey, phytosociology, literature review

## Abstract

Upland mires in Italy, excluding the Alps, have a fragmentary distribution, and most of them persist in climatically optimal mountain refugia. Based on the literature data, we assessed that the state of knowledge of Italian upland mires distributed in the Apennines, Sicily, and Sardinia is outdated. We analyzed 54 publications, and 220 peatland sites were found. Only a few publications were published in the last ten years, and most of the peat bogs described in the past have not been resurveyed. The largest number of sites is concentrated in the Tuscan-Emilian Apennines (60), followed by Sicily (51 sites) and Calabria (42). The vegetation belongs to 38 phytosociological associations, 19 sub-associations and variants, and 54 communities of 6 different classes; the most represented class is *Scheuchzerio palustris-Caricetea fuscae*. The most widespread disturbances are uncontrolled grazing by domestic livestock and wild fauna, groundwater extraction, and road construction. New investigations are urgently needed to update the state of Italian upland mires knowledge, which is the basis for all conservation strategies prescribed by national, European, and international policies.

## 1. Introduction

Peatlands are wetland environments formed through the accumulation of organic matter, where the rate of accumulation exceeds that of mineralization [1]. From coastal zones to the Alpine biome, various types of wetlands occur, such as marshes, swamps, and peat bogs, reflecting climatic, pedological, and hydrological variability [2,3].

In all cases, peatlands are characterized by permanently waterlogged soils, leading to low dissolved oxygen concentration. This hydrochemical condition slows down the decomposition of organic matter, promoting the accumulation of thick peat layers [4]. Several factors—including low nutrient availability, soil acidity, cold temperatures, and the acidifying effect of *Sphagnum* mosses—limit the growth and regeneration of woody species, favoring the establishment of a characteristic and low-productivity herbaceous vegetation [5,6]. Events such as lowering of water supply [7] and increasing nutrient load [8] are among the most common causes of peatland degradation.

Although peatlands cover only 3% of the Earth’s surface, they store a third of global soil carbon [9]. However, the balance between stored and emitted carbon is variable over time due to climate and environmental changes [10,11].

Peatlands are also relevant paleoenvironmental archives, storing past pollen assemblages [12].

From a legal point of view, peatlands’ restoration is in line with the recent Regulation (EU) 2024/1991 of the European Parliament and of the Council of 24 June 2024 on Nature Restoration and amending Regulation (EU) 2022/869, which forces the Member States to restore drained peat bogs (Art.11). Furthermore, strategies against peatland soil’s degradation respond to Goal 15.3 of the 2030 Agenda. Ramsar COP 14 (2022) also underlines the importance of acquiring peatland distribution information at a local scale. Finally, mires are protected by Directive 92/43/EEC through the designation of specific habitat types.

The aim of this review is to provide an updated synthesis of knowledge about Italian upland peatland’s vegetation through the analysis of the literature data. For peatland biotope, vegetation is a good indicator of abiotic factors’ variability, as demonstrated in some European regions [8]. For this reason, in our review, we have selected only studies that describe the typical mountain peaty communities, i.e., the oligotrophic vegetation that settles on substrates with high organic matter contents, more or less permanent water-satured, close to springs or at the edges of terestrialized water bodies.

### Peatlands in Italy

In the Mediterranean region, upland mires have a relict character for different reasons, like historical-genetic factors, given that their origin is linked to the effects of glacial periods on the natural landscape (e.g., most Tuscan-Emilian Apennines peat bogs develop on depressions whose origin can be traced back to the Quaternary glaciers’ melting [13]); biogeographic factors, hosting plant species with a N-European and Circumboreal typically centered distribution, many of which reach the southern limit in Mediterranean peat bogs [14,15,16,17,18,19]; and distributive factors, being present sporadically and discontinuously in mountain areas, often habitat-fragmented [20]. Consequently, they are isolated in climatically sub-optimal mountain refugia [21].

For these reasons, although their carbon sequestration rates are low compared to those of Northern Europe [22], Italian peatlands contribute to increasing environmental heterogeneity. In fact, due to their distinct characterized hydrological and geochemical conditions, they represent a refugia for specialized vegetation often associated with stenoecious microalgal communities [23]. The vegetation of Mediterranean upland peatlands, typically oligotrophic, is in many cases physiognomized by vascular plants of the genera *Carex* L., *Juncus* L., and *Eriophorum* L. associated with carpets and hummocks of bryophytes of the genera *Sphagnum* L., *Philonotis* Brid., *Sarmenthypnum* Tuom. & T.J. Kop., *Bryum* Hedw., *Calliergonella* Loeske, *Aulacomnium* Schwägr., *Polytrichum* Hedw., etc. The vegetation depends on site-specific abiotic factors’ variation, such as water origin and chemistry, soil wetting degree, and micromorphology [24].

In the Mediterranean region, peatlands are highly under-researched [22]. In Italy, out of a total land area of 302,073 km^2^ [25], the estimated peatland area is approximately 750 km^2^ [26,27].

On an altitudinal basis, three different types of peatlands can be recognized in Italy:

- Lowland type: coastal marshes, swamps, swamp forests, fens, lacustrine, and riverine mires that develop from sea level to the low hills. The vegetation is characterized by helophytes, which grow along the shores of water bodies, such as *Phragmites australis* (Cav.) Trin. ex Steud. Sometimes, patches of taller, drier vegetation (like tall sedge beds) develop from deeper water areas. This type of peatland was once the most widespread in Italy but has greatly declined over time due to peat cutting, land drainage for farming, and urban development [27]. Today, some of the last large lowland peatlands can still be found in the Veneto Po Valley [28], in the southern foothills of the Alps (Serbino mires, BS, [29]), and in the plains of northwestern Tuscany, including Massaciuccoli lake and marsh (PI, [30]) and the San Rossore swamp forest (PI, [31]).
- Alpine type: ombrotrophic acidic mires distributed throughout the Alpine arc, characterized by ombrotrophic *Sphagnum* hummocks alternating with species-poor carpets and pools, and minerotrophic fens [32]. The typical vegetation is represented by *Sphagnion medii* Kästner et Flössner 1933 Alliance (*Oxycocco-Sphagnetea* Br.-Bl. et Tx. ex Westhoff et al. 1946) for ombrotrophic peat bogs and *Scheuchzerion palustris* Nordhagen ex Tx. 1937 and *Caricion davallianae* Klika 1934 (*Scheuchzerio palustris-Caricetea fuscae* Tx. 1937) for minerotrophic fens.
- Upland type: fens and transitional mires found at mountain elevations. They are colonized by small sedges (*Caricion davallianae* Klika 1934), rush beds, swards (*Dactylorhizo-Juncion striati* S. Brullo et Grillo 1978), and, in some areas, by more oligotrophic communities with *Sphagnum* mosses (*Caricion fuscae* Koch 1926 *nom. conserv. propos.*). This type is different from alpine bogs in elevation, geographical distribution, water source (mainly saturated from mineral-rich groundwater), and vegetation structure and composition (generally higher species richness than alpine bogs [27]). Geology is the main factor influencing the development of mountain peatlands. In fact, fine-grained arenaceous soils reduce rainwater percolation [33] and trap groundwater, favoring mire development; in contrast, peat bogs are less likely to form in areas with very permeable soils [34].

## 2. Methods

### 2.1. Area of Study

**Figure 1:**
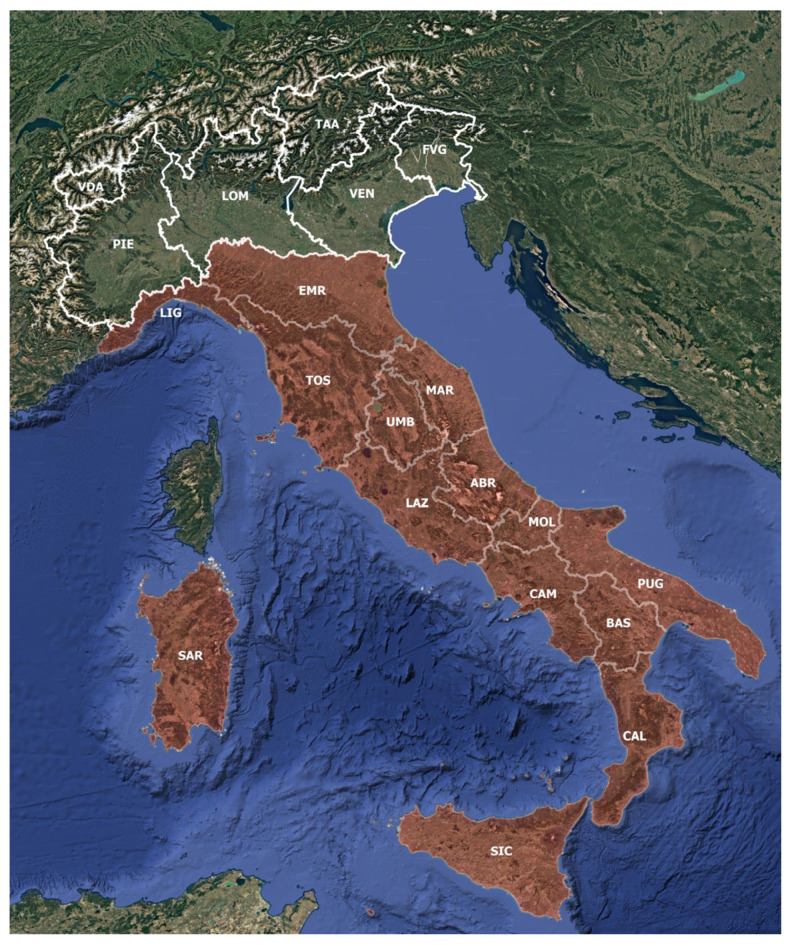
Delimitation of the area of study: the administrative regions under analysis are colored in red.

Our investigation was focused on peatland vegetation distributed in the mountainous belt of peninsular Italy, Sicily, and Sardinia (Figure 1). Studies concerning alpine and lowland peatlands were excluded. No studies were selected from Friuli Venezia Giulia (FVG), Lombardy (LOM), Piedmont (PIE), Valle d’Aosta (VDA), and Trentino Alto-Adige (TAA) administrative regions because in these areas, peatlands are located in the Alpine belt, whereas all the studies of Venetian (VEN) peatlands describe lowland environments, which are not the object of our study. In fact, as already discussed, alpine and lowland peatlands are structurally and physiognomically very different from upland peatlands, and they are located in very different bioclimatic contexts. Studies focused on nontypical peaty vegetation, i.e., marshy or lacustrine upland biotopes with tall sedge-bed vegetation (e.g., some communities of the *Magnocaricion elatae* Koch 1926 alliance), were not considered in this review.

Excluding the Alps, the Italian mountains are constituted in the peninsula by the Apennine chain, which extends for about 1000 km, from the Genoa area to the Sibari Plain, in Calabria, where it is in contact with the Calabro-Peloritano Arc (Sila, Serre, Aspromonte, Peloritani). These are mountains with altitudes ranging from 800 to 2000 m a.s.l., with few peaks exceeding 2000 m a.s.l. On a latitudinal basis, the Apennine chain is subdivided, from N to S, into Northern Apennines, Central Apennines, and Southern Apennines. Beyond Mount Cimone (2165 m a.s.l.), in the Tuscan-Emilian Apennines, the area with the highest altitudes is the central Apennines (Gran Sasso 2912 m a.s.l.; Maiella 2793 m a.s.l.). In the largest island, Sicily, apart from the Peloritani Mountains—which represent a strip of the Calabrian-Peloritan Arc—and the Iblei Mountains, the Madonie, the Nebrodi, the Palermo Mountains, and the Sicani Mountains belong to the “Maghrebides chain”, a mountain range geologically connected to North Africa. The mountains of the second largest island, Sardinia, are not genetically linked to those of the peninsula, and in altitudinal order, they are, respectively, the Gennargentu massif, with the 1834 m a.s.l. of Punta La Marmora, the Supramonte of Oliena, with the 1463 m a.s.l. of Punta Corrasi, and Limbara, with the 1362 m a.s.l. of Punta Sa Berritta [35].

### 2.2. Literature Analysis

All published studies concerning biological and environmental aspects (floristics, vegetation, and ecology) of Italian upland peatlands were analyzed (Table S1). For the bibliographical research, the Habitat Italia website, http://vnr.unipg.it/habitat/ (accessed on 10 February 2025), which provides a list of local publications for each 92/43/CEE Directive Habitat type, was used, and, after that, keyword research using the Google Scholar search engine was performed. The types of information derived from the publications were classified in “Topics” (Table 1). Spatial information was resumed from each study, i.e., the administrative regions in which the studied sites fall and the presence of precise geographical references (“Published geographic coordinates”) or, in case of absence of geographical coordinates, it was reported whether the described sites could be easily found with known local references (“Local reference individuable”), like many high-altitude lakes.

Publications that simply include single plant species reports (floristic reports and notulae) were excluded, except those that provide detailed information on the site’ ecological features (e.g., [14]). In fact, although some vascular plants, ferns, or bryophyte species are indicators of typical peatland abiotic conditions, it was preferred to give greater attention to studies that describe peat bogs from a broader point of view, revealing environmental, ecological, and vegetational aspects. In fact, in most cases, floristic reports, especially the oldest ones, contain vague spatial references or obsolete local toponyms, which are difficult to identify in the field. Furthermore, because Italian upland peat bogs are highly fragmented and scattered, it is common to find small, isolated populations of characteristic plant and bryophyte species even in areas where typical peatland conditions are no longer present. Conversely, vegetation studies typically provide more detailed information on the distribution, structure, and composition of plant communities, including full phytosociological relevés.

Furthermore, in order to provide a measure of the national and regional knowledge updating, we considered “recent publications” those published in the last 10 years; therefore, those prior to 2015 are considered “old publications”. This temporal interval was chosen because the terestrialization dynamics of peat bogs in the Mediterranean region are very rapid, since most of them accumulate any more peat [36]. This interval is also close to that prescribed by the Italian Interpretation Manual of Directive 92/43/EEC Habitats for updating the cartography of 7140 Habitat [37].

### 2.3. Peatland Sites Inventory: Regional Distribution and Vegetation

All the peat bog sites described in each publication were examined. The main criteria for site selection was the vegetation, since only sites with typical upland peatland plant communities [38] were selected and listed in a table (Table S2). Each site has been reported with its original name (“Toponym”), to which the administrative region (“Administrative Region”) and, when possible, the province (“Province”) and the municipality (“Municipality”) have been associated. When available, the published geographic coordinates have been reported for each site, and an indication of the vegetation type (“Plant communities”) has also been included. Furthermore, for all peat bog sites, the most recent studies confirming their presence in optimal condition or declaring their extinction have been reviewed.

To represent plant communities variability, we summarized all the published phytosociological associations and communities in a syntaxonomical scheme (Appendix A). The syntaxonomical nomenclature of class, orders, and alliances follows [38], while that of vascular plant and bryophyte *taxa* cited in the article follows [39,40] respectively.

Finally, we assessed how many sites with known precise locations, that is, those that are georeferenced or have identifiable local references, are located within protected areas. These include Ramsar sites, national parks, regional parks (including state biogenetic reserves), and sites protected under Directive 92/43/EEC and Directive 2009/147/EC (ZPS, SIC, and ZSC).

## 3. Results

### 3.1. Literature Analysis

**Figure 2:**
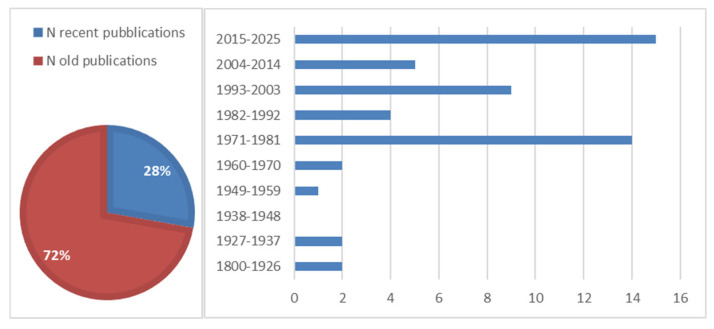
Temporal distribution of upland peatland publications on a national level (“recent publications” are studies published in the last 10 years).

From the analysis of the literature data, it resulted that 54 are the specialist studies on Italian upland mires. In total, 72% (39) of the studies were published before 2015 (“old publications”); only 28% (15) of the studies were published in the last 10 years (“recent publications”) (Figure 2). The oldest publication dates back to 1817, while the most recent was published in 2024. The highest number of publications for a 10-year interval was produced in the 2015–2025 (15) and 1981–1971 (14) intervals, while the decades between 1927 and 1970 record the lowest number of published studies.

**Figure 3:**
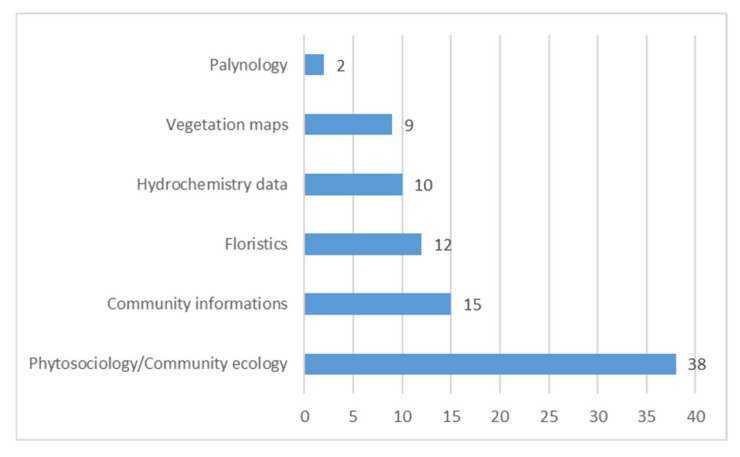
Number of publications per topic.

Concerning the type of information (“Topics”) reported, 38 publications present published phytosociological relevés, 15 contain only descriptions of the plant communities’ composition but not quantitative vegetational data, 9 publications are also accompanied by vegetation maps, while 12 report floristic data (checklists); only in 10 studies were soil/water hydrochemical variables measured, and finally 2 present palynological data (Figure 3 and Table S1).

Out of the total number of studies, only 14 of them report published geographical coordinates of the described sites (Table S1).

**Figure 4:**
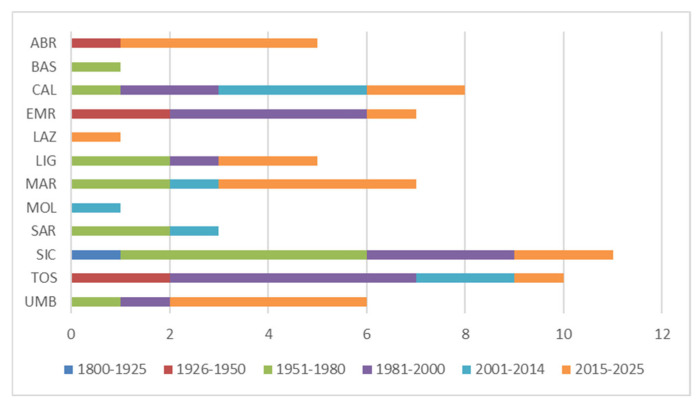
Temporal distribution of upland peatland studies on a regional level (ABR = Abruzzo; BAS = Basilicata; CAL = Calabria; EMR = Emilia-Romagna; LAZ = Lazio; LIG = Liguria; MAR = Marche; MOL = Molise; SAR = Sardinia; SIC = Sicily; TOS = Tuscany; UMB = Umbria).

Regarding regional distribution of studies, the largest number of studies comes from the Sicily (SIC, 11), Tuscany (TOS, 10), and Calabria (CAL, 9) administrative regions, even if they are mostly old studies (Figure 4), while the least studied regions are Basilicata (BAS), Lazio (LAZ), and Molise (MOL), represented by only 1 recent study; Campania (CAM) and Puglia (PUG) do not even include any publication. In Abruzzo (ABR), Marche (MAR), Umbria (UMB), and Lazio (LAZ), the largest number of recent studies is concentrated (Figure 4 and Table S1).

### 3.2. Peatland Sites Inventory: Regional Distribution and Vegetation

**Figure 5:**
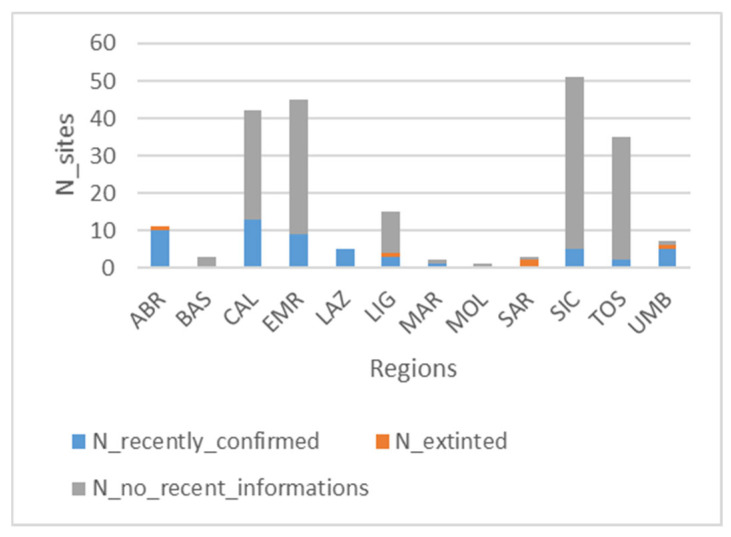
Level of knowledge updating on a regional level (blue: number of recently confirmed sites; grey: number of sites not recently confirmed; and orange: number of extinct sites).

A total of 220 sites were identified. Only 61 of them are georeferenced, while 33, although not georeferenced, are identifiable on the territory because they are located in proximity to known local references (“local reference individuable”, Table S2). About sites recently confirmed, it results that only 53 sites are still present in optimal conditions, as 36 of them have been recently described, while the remaining 17 were studied in the past, and they have been reconfirmed in the last 10 years; 5 sites have been declared extinct, while for the remaining 162 sites, there is no recent information on their actual existence. The administrative region with the highest number of studied sites is Sicily (51), with 46 sites no longer confirmed and 5 recently surveyed (Figure 5), followed by Emilia-Romagna (45), Calabria (42), and Tuscany (35), while those with the least number of sites are Lazio, Basilicata, Marche, Sardinia, Molise, and Umbria (Figure 5 and Table S2).

Out of 94 georeferenced and “local reference individual sites”, only 3 are outside protected areas (Table S2). Additionally, our analysis shows that among these sites, 53 have not been recently confirmed.

The sites under analysis are colonized by a wide range of plant communities, from typical *Scheuchzerio palustris-Caricetea fuscae* Tx. 1937 communities to fen grasslands with many *Molinio-Arrhenatheretea* Tx. 1937 more generalist species (Table S2). A total of 38 phytosociological associations, 19 sub-associations and variants, and 54 communities belonging to 6 classes were recognized (Appendix A). The *Scheuchzerio palustris-Caricetea fuscae* Tx. 1937 includes most of the associations and communities (65%), followed by *Molinio-Arrhenatheretea* Tx. 1937 (16%), *Phragmito-Magnocaricetea* Klika in Klika et Novak 1941 (11%), *Montio-Cardaminetea* Br.-Bl. et Tx. ex Klika et Hadač 1945, *Littorelletea uniflorae* Br.-Bl. et Tx. ex Westhoff et al. 1946, and *Oxycocco-Sphagnetea* Br.-Bl. et Tx. ex Westhoff et al. 1946. Higher *syntaxa* are summarized here. *Littorelletea uniflorae* class in Italy includes amphibious plant vegetation developed on peaty substrates permanently saturated by water (*Hyperico elodis-Sparganion* Br.-Bl. et Tx. ex Oberd. 1957, *Littorellion uniflorae* Koch ex Klika 1935 alliances); the *Molinio-Arrhenatheretea* includes the relict humid fen grasslands of the southern upland belts of the Italian peninsula and Sicily (*Dactylorhizo-Juncion striati* S. Brullo et Grillo 1978, *Calthion palustris* Tx. 1937), the mown wet meadows of siliceous Apennines highland (*Molinio-Holoschoenion* Br.-Bl. ex Tchou 1948, *Molinion caeruleae* Koch 1926) and the wet meadows of high-altitude karst poljes of the Central Apennines (*Ranunculion velutini* Pedrotti 1978); besides, the *Montio-Cardaminetea* class describes the oligothrophic vegetation of peaty springs in mountain clearings (*Cardamino-Montion* Br.-Bl. 1926) or in shady forest environments (*Caricion remotae* Kastner 1941) and also the vegetation of moss-rich calcareous water springs (*Cratoneurion commutati* Koch 1928); *Scheuchzerio-Caricetea* groups the sedge-moss calcareous mineral-rich fen vegetation (*Caricion davallianae* Klika 1934) or the transitional-mires’ vegetation (*Caricion fuscae* Koch 1926), while only one association of Tuscan-Emilian Apennines [16] represents *Oxycocco-Sphagnetea* in Apennines context; *Phragmito-Magnocaricetea* includes the helophyte, sedge and rushes bed, and herbland vegetation of the most advanced successional stages of mires lifting.

## 4. Discussion

### 4.1. State of Knowledge

As it is known, mires in the Mediterranean region are particularly understudied [22]. In fact, our results demonstrate that the state of knowledge on upland peatland vegetation in Italy is very scarce, given that 72% of the studies are old and 74% of the sites have not been reconfirmed in recent years.

Until 1970, the number of studies per decade was very low, while there is a noteworthy increase in the 1971–1981 decade, probably coinciding with the spread of a general interest in nature conservation [41].

The lack of precise geographical references in many studies, especially the oldest ones, is a significant issue at the national level, as only 27% of the sites are georeferenced. This fact makes it impossible to relocate and resurvey historically described peat bogs unless additional information is obtained from local experts.

Italian mountain peatlands are not always individual through aerial photographs, since they are often small, fragmented, and closely surrounded by forest or grassland vegetation [14,20,42,43,44,45]. However, the lack of knowledge about peatlands in Italy is partly due to a broader issue: the decline in the number of specialists in peatland science, particularly in regions where peat bogs are rare [22]. Moreover, most studies include phytosociological relevés, which could help check how plant communities have evolved over time if the same locations are surveyed again. The plot size in Italian studies varies depending on the structure of plant communities, and it does not always align with that recently suggested at the European level by [46], who proposed that the optimal plot size for fens and transitional mires (*Scheuchzerio-Caricetea fuscae*) and bogs (*Oxycocco-Sphagnetea*) is equal to 16 sqm. For example, [16] apply large survey areas (10–30 sqm.) for sedges (*Caricetum nigrae* Br. Bl. 1915) and smaller plots (1–5 sqm.) to detect bryophyte-dominated communities (*Sphagno-Caricetum nigrae* Gerdol & Tomaselli 1993 and variants), whose variability depends on fine-scale factors, like micromorphology and interspecific competition [47].

**Figure 6:**
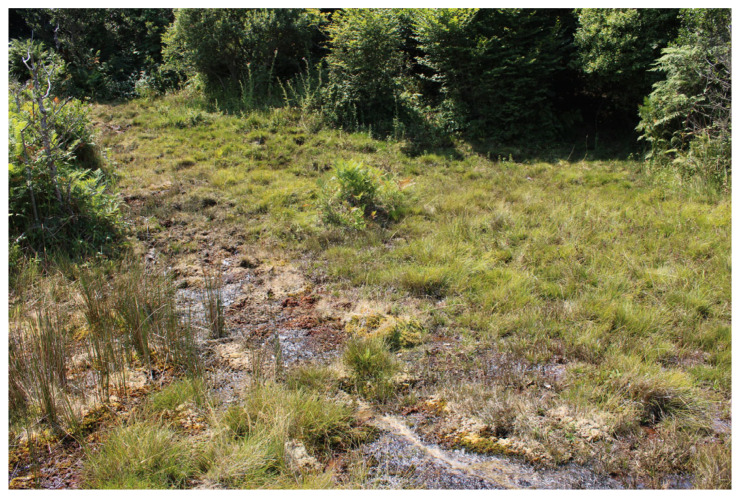
The unique *Sphagnum* mire known for Sicily (Madonie mountains, PA). It is covered by a lawn of *Carex echinata* Murray, *Juncus* sp. pl., carpets of *Sphagnum* sp. pl. alternating with small *Polytrichum commune* Hedw., *Aulacomnium palustre* (Hedw.) Schwägr., and *Osmunda regalis* L. hummocks.

Regarding the regional distribution of sites, the number of recorded sites does not always reflect regional knowledge updating. In fact, in Sicily, of the 51 studied sites, 46 of them were described in the years 1978–1980 [17,48,49] and 1994 [50] and never resurveyed in recent studies, while only 5 peat bogs have been recently confirmed: one of them is the unique *Sphagnum*-peat bog known on the island (Madonie, Figure 6) [42], while the others, distributed on the Nebrodi mountains, are fen grasslands of *Thelypterido palustris-Caricetum paniculatae* Sciandrello, Cambria, Giusso, Tavilla, Minissale 2021. The other Sicilian peat bogs, no longer confirmed, are transitional mires of *Caricion fuscae* (Madonie, [17,48]) and fen grasslands rich in species, dominated by sedges and rushes (Nebrodi, *Dactylorhizo-Juncetum effusi* Brullo & Grillo 1978, *Caricetum intricato-oederi* Brullo & Grillo 1978) alternating with helophytic plant communities (*Glycerio spicatae-Oenanthetum aquaticae* Brullo, Minissale & Spamp. 1994, *Oenantho fistulosae-Glycerietum spicate* Brullo and Grillo 1978).

**Figure 7:**
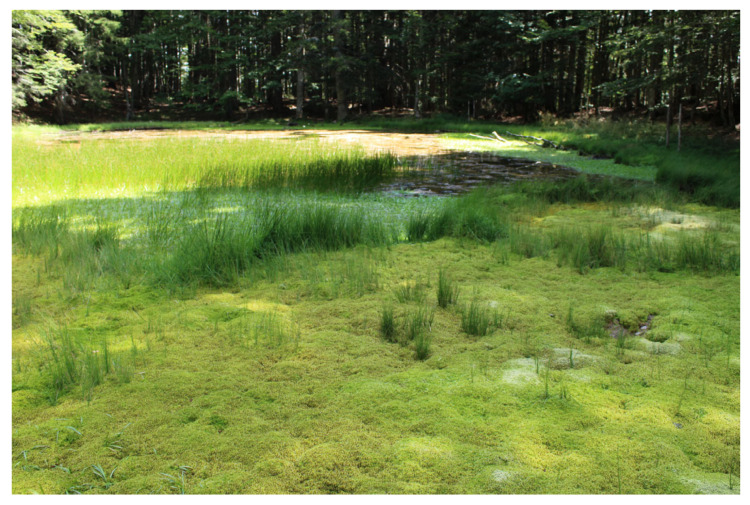
The lacustrine mire developed around the shores of the Greppo lake (Tuscan-Emilian Apennines, Tuscany, PT). Vegetation zonation is well developed with the *Sphagnum* carpet around the shore and *Sparganium natans* L. colonizing the floating zone back to the transitional drier facies dominated by *Carex echinata* Murray and *Agrostis canina* L.

**Figure 8:**
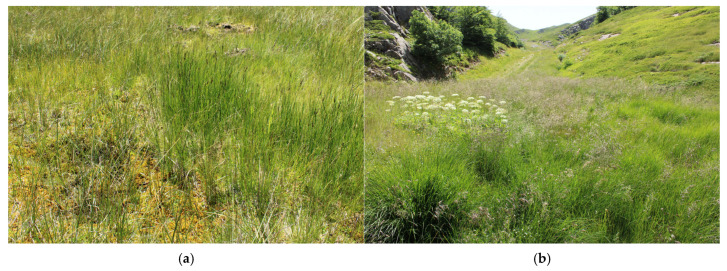
(**a**) Transitional mire characterized by typical species of *Caricetum nigrae* (Tuscan-Emilian Apennines, Tuscany, PT) and (**b**) a drier facies of the same site colonized by high-productive grasses of the *Molinio-Arrenatheretea* class (*Deschampsia cespitosa* (L.) P.Beauv. and *Molinia caerulea* (L.) Moench).

The largest number of described peat bogs is concentrated in the Tuscan-Emilian Apennines (60 sites), a section of the northern Apennines that spans the border between Emilia-Romagna and Tuscany. In this area, peat bogs predominantly develop on the Adriatic-facing backslopes, which are less steep than the Tyrrhenian side. These peat bogs typically fill depressions formed by periglacial processes—such as nivation niches, nival moraines, and gullies—that began with post-glacial melting and continued throughout the Holocene [13,16,51]. The peatlands of this area include not only acidic peat bogs, mountain springs, and fen grasslands but also lacustrine mires (Lago Nero, Lago del Greppo, etc.) (Figure 7); they are mostly characterized by vegetation of the *Scheuchzerio-Caricetea fuscae* Class (*Caricetum nigrae*) (Figure 8a), which in many cases tends to evolve towards *Molinio-Arrhenatheretea* fen grasslands [51,52] (Figure 8b). This Apennines sector supports vegetation types and plant species with a N-European and Circumboreal typically centered distribution; notably, it hosts the only known occurrence of the *Oxycocco-Sphagnetea* class—*Sphagnetum magellanici* (Malcuit 1929) Kästner et Flössner 1933—outside the Alpine region. Several vascular plant species also reach their southern distribution limit here, i.e., *Swertia perennis* L., *Gentiana asclepiadea* L., *Eriophorum scheuchzeri* Hoppe, *Eriophorum angustifolium* Honck., and *Trichophorum alpinum* (L.) Pers. [39,53]. The only Tuscan peat bogs outside the Tuscan-Emilian Apennines are located in the Apuan Alps (LU): these are alkaline fens with *Caricion davallianae* typical species and fen grassland rich in *Molinio-Arrhenatheretea* species [45,54]; however, no recent studies have reconfirmed their persistence.

Then, the peat bogs described for Calabria are transitional *Sphagnum*-mires (*Scheuchzerio palustris-Caricetea fuscae*), relict humid swards (*Dactylorhizo-Juncion striati*), fen grasslands (*Molinio-Arrhenatheretea*), and peat bogs permanently saturated by water (*Littorelletea uniflorae*) [18]. Most of the *Sphagnum*-mires of Calabria, distributed in the Aspromonte massif, have been resurveyed by a recent study [55].

The alkaline peaty meadows of Pollino, which are the only mires in the Basilicata region, have not been confirmed since the first publication [56].

Instead, some of the peat bogs described for Liguria are distributed on the Piedmont-Ligurian Apennines [57], others occur in the Genova province, inside the Agoraie-protected area [43,58]; their vegetation is similar to that of the Tuscan-Emilian Apennines’ mires, being represented mostly by the *Caricion fuscae* and *Molinion caeruleae* alliances.

In the Central Apennines (Abruzzo, Marche, Umbria, and Lazio), the vegetation of alkaline peat bogs (*Caricion davallianae*) finds refuge in the karst plateau system, covering very little area [44,59,60], while the unique peat bog known in Molise, i.e., the “Pantano della Zittola”, has not been studied recently. However, despite the Central Apennines presenting the greatest number of recent studies (Abruzzo, Marche, Umbria, and Lazio; Figure 4), the peat bogs described from these regions are quite few compared to the national distribution (Figure 5).

About Sardinia mires (3), of those initially reported by [14], two of them have been declared extinct [61], while the presence of the already known last *Sphagnum* population is doubtful.

To plan effective long-term conservation for species and habitats, it is essential to update the status of Italian upland mires, particularly those that fall within protected areas.

### 4.2. Disturbance, Pressures, and Threats

Mediterranean peatlands are themselves located at the climatic limit for peat formation [22,36]; consequently, they are potentially more vulnerable to degradation mediated by climate change [62] and anthropogenic disturbances. While land reclamation for agriculture is the most common threat to lowland peatlands in Europe [4], upland peatlands face different pressures, which vary regionally across Italy. This is largely due to the highly heterogeneous geology of the Italian mountains, which has historically influenced patterns of land use [63]. Additionally, climatic variability on a latitudinal gradient affects locally the type and seasonality of human activities—such as livestock farming, agriculture, and tourism—which in turn impact mountain ecosystems in diverse ways. In some cases, these human activities contribute to speeding up the territorialization process of peat bogs, which naturally occurs over a very long time and in *Sphagnum*-peat bogs is mediated by their vertical growth [17].

**Figure 9:**
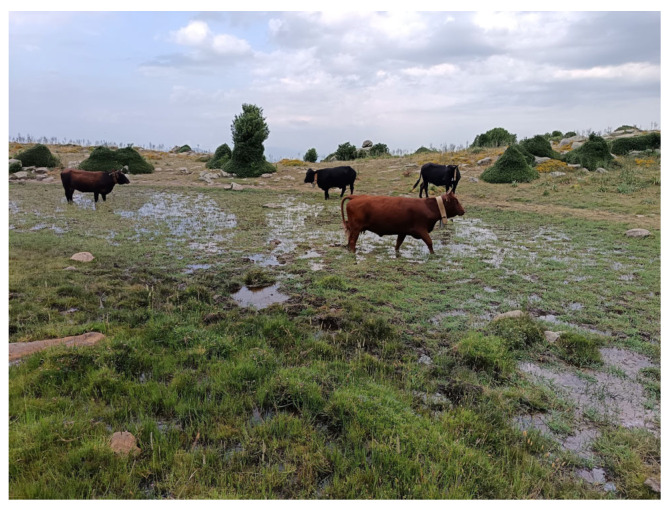
Uncontrolled grazing of cows in a Sicilian peat bog.

At the national level, the most common disturbance is uncontrolled grazing by domestic livestock (Figure 9), which leads to both physical damage from trampling and chemical alterations due to nutrient enrichment from dump deposition [17,44,64]. Wild fauna (wild boars, European fallow deer, and other ungulates) cause damages similar to domestic livestock [55]. Other common disturbances are road construction, which causes not only local water regime alterations [17,57,61] but also the groundwater extraction for agriculture or to supply mountain villages [17,55]. According to the III and IV Italian Report of Habitat Directive (2007–2012 and 2013–2018), the variation in water regimes is the type of pressure—i.e., the actions or factors that have acted in the past, in the last 6 years, and/or that are still active [65]—that has the greatest negative impact on the mires’ 92/43/CEE Directive Habitats.

**Figure 10:**
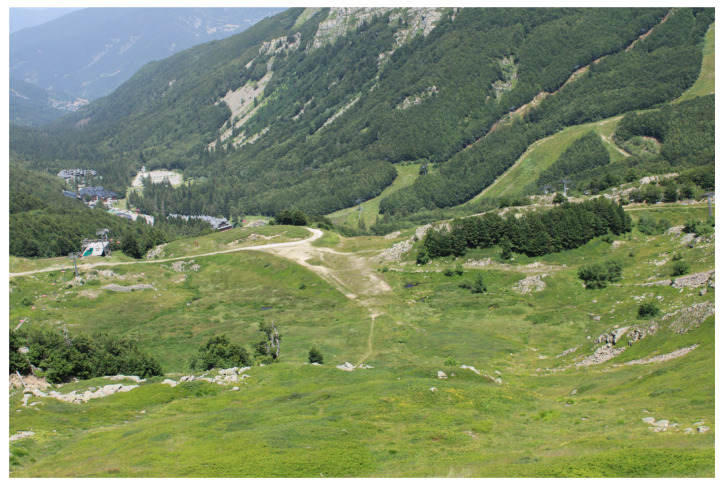
The peat bog in the foreground was previously a large homogenous site before being fragmented by an artificial mountain path (Tuscan-Emilian Apennines, Tuscany, PT).

It is common for sites to be affected by multiple types of pressures over time. For example, in a large peat bog in the Abruzzo administrative region, several multiple pressures have led to the regression of peaty vegetation [66], i.e., peat extraction for productive purposes, which ceased in 1950, and subsequently other anthropic activities (drainage and planting of willows and poplars). Furthermore, several peatland alterations are also caused by uncontrolled tourism and recreational activities; for example, in Val di Luce (PT), an area of the Tuscan-Emilian Apennines, some mires have been spatially fragmented due to the opening of new ski slopes or passages for motocross races, as shown in Figure 10 [51,52].

Some mountain areas are naturally inhospitable for mire development, e.g., the Apuan Alps, a Tuscan limestone massif whose steep slopes and dominant calcareous bedrock make surfaces extremely permeable; consequently, the rare flat humid sites have often been converted to livestock pastures [34].

A particularly significant case of mire degradation due to anthropogenic causes is that of the Palude di Colfiorito (PG) in Umbria [67]. It is a biotope originally consisting of a system of natural wet depressions: pools (*Charion intermediae* Sauer 1937, *Nitellion flexilis* W. Krause 1969, *Potamogetonion* Libbert 1931), marshes (*Phragmition communis* Koch 1926), fens (*Caricion davallianae*), and springs (*Glycerio-Sparganion* Br.-Bl. et Sissingh in Boer 1942). However, compared to the ecological status reported by [59], morphological changes induced by man have occurred over the years, which have led to the extinction of the typical peaty plant communities. In 1980, in fact, deep trenches were dug around the peat bog area, causing drainage in a few years and consequently the disappearance of the pre-existing peatland phytocenoses. The drained area was colonized by many ruderal species, i.e., *Conium maculatum* L., *Dipsacus fullonum* L., *Pastinaca sativa* L. *subsp. urens* (Req. ex Godr.) Čelak., and a hygrophilous herbaceous vegetation with dominance of *Deschampsiacespitosa* (L.) P. Beauv. and *Agrostis stolonifera* L. established later. Afterwards, the secondary succession went on with the establishment of shrubby species, i.e., *Rhamnus cathartica* L. In the case of Colfiorito, other anthropogenic multiple stressors have modified the entire biotope, such as the digging of channels and the planting of a large poplar grove, peat extraction activities, water extraction, and clearing and plowing of wet meadows. However, anthropic activities do not always cause negative impacts on peat bog development, for example, periodic mowing and controlled grazing could slow down, in some cases, the territorialization process, preventing the rapid invasion of shrub communities [59,68].

### 4.3. Upland Peatland Interest in Italy: From Exploitation to Conservation

In Italy, the interest in peatland has changed over time. In fact, from the industrial revolution to the first decades of the 20th century, the search for peat bog sites, especially lowland ones, was promoted by exploitation interests, since in that period peat was used as fuel for domestic heating, furnaces, mills, factories, and, in some cases, to power trains [27]; for agricultural purposes as litter or fertilizer; and for industrial uses, it could be transformed by distillation into many products (ammonium sulfate, tar, heavy oils, paraffin, and alcohol). However, peatland exploitation decreased around the middle of the 20th century [69], probably due to the rise of a nature conservation interest. In fact, the few exploitation attempts made after this period were largely prevented by conservation regulations [67].

To date, Directive 92/43/EEC, implemented with D.P.R. 8 September 1997 n. 357, amended and integrated by D.P.R. 12 March 2003 n. 120, is the main law that regulates the monitoring and conservation measures of mires within the areas protected by 92/43/EEC Directive (SIC, ZPS, and ZSC) in Italy. The habitats related to mountain Italian mires are 7110* Active raised bogs; 7120 Degraded raised bogs still capable of natural regeneration; 7140 Transition mires and quaking bogs; 7150 Depressions on peat substrates of the *Rhynchosporion*; 7210* Calcareous fens with *Cladium mariscus* and species of the *Caricion davallianae*; 7220* Petrifying springs with tufa formation (*Cratoneurion*); 7230 Alkaline fens; 6420 Mediterranean tall humid herb grasslands of the *Molinio-Holoschoenion*; and 6410 Molinia meadows on calcareous, peaty, or clayey-silt-laden soils (*Molinion caeruleae*). However, Habitat 7110* Active raised bogs represents *Sphagnion magellanici* communities (*Oxycocco-Sphagnetea* Class), which are found exclusively in the form of very rare populations in the Tuscan-Emilian Apennines [16], while they are much more widespread in the Alps.

The interpretation of Italian upland mires under the 92/43/EEC Habitat Directive is not always easy and straightforward. In fact, the EUR 28 Manual defines habitats based on both vegetation composition and abiotic factors, such as water and soil chemistry. However, studies from the Laga Mountains [33] and the Tuscan-Emilian Apennines [70] have shown that some small peat bogs are colonized by alkaline fen communities of *Caricion davalliane*, which are typically associated with Habitat 7230, even though they settle on acidic soils, contradicting the original diagnosis in the EUR 28 Manual. Furthermore, certain mountainous biotopes, such as slightly acidic and acidic fen-springs hosting *Cardamino-Montion* and *Caricion remotae* communities (see Appendix A), do not clearly fit any recognized habitat of the EUR 28 Manual. For these reasons, a more in-depth survey of these biotopes at the national level is essential. All the information on habitats, regional distribution, and their conservation status is reported on the Habitat Italia website (http://vnr.unipg.it/habitat/, accessed on 10 February 2025).

Improving the classification and interpretation of habitat types in Italy is essential not only for the conservation of rare biotopes but also for refining estimates of the national extent of mires. As shown by [71], the distribution of peatlands in Europe is predominantly concentrated in northern regions, reflecting broader temperature and precipitation gradients. Among southern European countries (Albania, Andorra, Bosnia and Herzegovina, Croatia, Cyprus, Greece, Italy, Montenegro, Portugal, Republic of Macedonia, Serbia, Slovenia, Spain, and Turkey), Italy ranks third in estimated mire area, with approximately 120 km^2^, following Spain (200 km^2^) and Bosnia and Herzegovina (162.5 km^2^) [71]. However, this figure more accurately reflects the extent of habitat vegetation cover, which does not necessarily correspond to the full surface area of Italian mires. Therefore, it is likely an overestimation [27].

## 5. Conclusions and Future Directions

Our study assessed that the state of knowledge of upland mires in Italy is outdated, as it is for most countries in Europe. This phenomenon could lead to a progressive loss of information on local distribution of peat bogs that is the starting point for future conservation and restoration strategies prescribed by national, European, and international policies.

Italian upland mires have a fragmentary distribution, and most of them persist in mountain climatical refugia. These particular biotopes hold a high biogeographic value in Italy given that they are the southernmost *Sphagnum* mires in Europe. These biotopes and related plant specialists have already suffered habitat reduction and fragmentation from several anthropogenic pressures, and they could be imperiled by the effects of climate change. Acquiring vegetation, floristics, and environmental information from field surveys is still fundamental for peatland conservation, as observed for other Mediterranean wetland biotopes [72]. As a consequence, updating Italian upland mire knowledge is urgently needed.

## Figures and Tables

**Table 1:** Classification of publications based on types of information and data reported (“Topics”).

Topics	Description
Floristics	Floristic lists and checklists
Community information	Vegetational qualitative information, only physionomic-like descriptions but not quantitative vegetational data
Phytosociology/Community ecology	Vegetation quantitative data (phytosociological relevés)
Vegetation maps	Vegetation maps
Hydrochemistry data	Abiotic variables of soil and circulating water
Palynology	Palynological data

## Data Availability

The original contributions presented in this study are included in the article/Supplementary Material. Further inquiries can be directed to the corresponding author.

## Supplementary Materials

The following supporting information can be downloaded at: https://www.mdpi.com/article/10.3390/plants14131931/s1, Table S1: Literature analysis; Table S2: Sites inventory: regional distribution and vegetation.

## Appendix A

### Syntaxonomical Scheme

LITTORELLETEA UNIFLORAE Br.-Bl. et Tx. ex Westhoff et al. 1946

 LITTORELLETALIA UNIFLORAE Koch ex Tx. 1937

  HYPERICO ELODIS-SPARGANION Br.-Bl. et Tx. Ex Oberd. 1957

  *Ranunculo fontani-Potametum polygonifolii* Brullo, Scelsi & Spampinato 2001

  LITTORELLION UNIFLORAE Koch ex Klika 1935

  *Carici demissae-Potametum polygonifolii* Gargano, Passalacqua & Bernardo 2007

  *Carici demissae-Potametum polygonifolii* Gargano, Passalacqua & Bernardo 2007

  *menyanthetosum trifoliatae*

MOLINIO-ARRHENATHERETEA Tx. 1937

 HOLOSCHOENETALIA Br.-Bl. ex Tchou 1948

  DACTYLORHIZO-JUNCION STRIATI S. Brullo et Grillo 1978

  *Agrostio aspromontanae-Juncetum bulbosi* Brullo, Scelsi & Spampinato 2001

  *Caricetum intricato-oederi* Brullo & Grillo 1978

  *Caricetum intricato-oederi* Brullo & Grillo 1978 *caricetosum oederi*

  *Caricetum intricato-oederi* Brullo & Grillo 1978 *caricetosum intricatae*

  *Caricetum stellulato-oederi* Brullo, Scelsi & Spampinato 2001

  *Dactylorhizo-Juncetum effusi* Brullo & Grillo 1978

  *Galio debilis-Deschampsietum caespitosae* Brullo, Scelsi & Spampinato 2001

  *Galio debilis-Deschampsietum caespitosae* Brullo, Scelsi & Spampinato 2001 *Genista an*

  *glica* variant [Gargano, Passalacqua & Bernardo 2007]

  *Holoschoeno-Caricetum distantis* Brullo, Scelsi & Spampinato 2001

  MOLINIO-HOLOSCHOENION Br.-Bl. ex Tchou 1948

  *Juncus subnodulosus* community [Venanzoni & Gigante 2000]

  *Molinietum arundinaceae* Trinajstic 1964

 MOLINIETALIA CAERULEAE Koch 1926

  CALTHION PALUSTRIS Tx. 1937

  *Chaerophyllo calabrici-Calthetum* Venanzoni 1988

  *Geranio asphodeloidis-Caricetum paniculatae* Venanzoni 1988

  *Scirpetum sylvatici* Ralski 1931

  MOLINION CAERULEAE Koch 1926

  *Deschampsia caespitosa* community [Foggi, Gennai, Gervasoni, Ferretti, Viciani, Venturi 2007]

  *Junco-Molinietum caeruleae* Preisg. 1951

 TRIFOLIO-HORDEETALIA Horvatić 1963

  RANUNCULION VELUTINI Pedrotti 1978

  *Deschampsio-Caricetum distantis* Pedrotti 1967 *eriophoretosum*

MONTIO-CARDAMINETEA Br.-Bl. et Tx. ex Klika et Hadac 1944

 MONTIO-CARDAMINETALIA Pawłowski et al. 1928

  CARDAMINO-MONTION Br.-Bl. 1926

  *Montio-Philonotidetum fontanae* Buker & R. Tx. In Buker 1942

  *Veronico beccanbugae-Ranunculetum ophioglossifolii* Brullo, Scelsi & Spampinato 2001

  CARICION REMOTAE Kastner 1941

  *Carici remotae-Osmundetum regalis* Brullo, Scelsi & Spampinato 2001

  CRATONEURION COMMUTATI Koch 1928

  *Cratoneuretum commutati* Aichinger 1933

OXYCOCCO-SPHAGNETEA Br.-Bl. et Tx. ex Westhoff et al. 1946

 SPHAGNETALIA MEDII Kästner et Flössner 1933

  SPHAGNION MEDII Kästner et Flössner 1933

  *Sphagnetum magellanici* (Malcuit 1929) Kästner et Flössner 1933

PHRAGMITO-MAGNOCARICETEA Klika in Klika et Novak 1941

 MAGNOCARICETALIA Pignatti 1953

  MAGNOCARICION ELATAE Koch 1926

  *Carex rostrata* community [Gargano, Passalacqua & Bernardo 2007]

  *Caricetum rostratae* (Dagys 1932) Bal.-Tul. 1963

  *Caricetum rostratae* Rubel 1911

  *Caricetum vesicariae* Br. Bl. et Den. 1926

  MAGNOCARICION GRACILIS Géhu 1961

  *Thelypterido palustris-Caricetum paniculatae* Sciandrello, Cambria, Giusso, Tavilla, Mi

  nissale 2021

 OENANTHETALIA AQUATICAE Hejny ex Balatova-Tulackova et al. 1993

  ALOPECURO-GLYCERION SPICATAE S. Brullo et al. 1995

  *Glycerio spicatae-Oenanthetum aquaticae* Brullo, Minissale & Spamp. 1994

  *Oenantho fistulosae-Glycerietum spicate* Brullo & Grillo 1979 *drepanocladetosum*

  *Oenantho fistulosae-Glycerietum spicate* Brullo e Grillo 1978 *glicerietosum spicatae*

 PHRAGMITETALIA Koch 1926

  PHRAGMITION COMMUNIS Koch 1926

  *Carex rostrata* community [Gerdol & Tomaselli 1993]

  *Menyanthes trifoliata* community [Gerdol & Tomaselli 1993]

  *Menyanthes trifoliata* community [Gargano, Passalacqua & Bernardo 2007]

  *Ranunculus flammula-Juncus articulatus* community [Gargano, Passalacqua, Bernardo 2007]

SCHEUCHZERIO PALUSTRIS-CARICETEA FUSCAE Tx. 1937

 CARICETALIA DAVALLIANAE Br.-Bl. 1950 nom. conserv. propos.

  CARICION DAVALLIANAE Klika 1934

  *Blysmus compressus* and *Juncus depauperatus* community [Bonin 1972]

  *Blysmus compressus* community [Ciaschetti, Praleskouskaya, Venanzoni 2024]

  *Carex acuta* and *C. panicea* community [Di Pietro, Praleskouskaya, Aleffi, Di Pietro, Di Pietro, Tondi, Fortini 2024]

  *Carex davalliana* and *C. echinata* community [Di Pietro, Praleskouskaya, Aleffi, Di Pietro, Di Pietro, Tondi, Fortini 2024]

  *Carex davalliana* and *C. hostiana* community [Di Pietro, Praleskouskaya, Aleffi, Di Pietro, Di Pietro, Tondi, Fortini 2024]

  *Carex echinata* community [Pedrotti & Murrja 2020]

  *Carex frigida* and *Juncus articulatus* community [Di Pietro, Praleskouskaya, Aleffi, Di Pietro, Di Pietro, Tondi, Fortini 2024]

  *Carex nigra* subsp. *nigra* community [Ciaschetti, Pirone & Venanzoni 2020]

  *Caricetum davallianae* Dutoit 1924

  *Caricetum davallianae* Dutoit 1924 *caricetosum hostianae* Ciaschetti, Praleskouskaya,

  Venanzoni 2024

  *Eleocharitetum quinqueflorae* Lüdi 1921

  *Eleocharitetum quinqueflorae* Lüdi 1921 *Carex oederi* and *Triglochin palustris* variant

  [Ciaschetti, Praleskouskaya, Venanzoni 2024]

  *Eriophoretum latifolii* Pedrotti 1967 nom. inval. ad interim

  *Eriophoretum latitolii* Pedrotti 1967 *cratoneuretosum commutati* Gerdol & Tomaselli

  1987 nom. inval. ad interim

  *Eriophorum latifolium* and *Carex frigida* community [Di Pietro, Praleskouskaya, Aleffi, Di Pietro, Di Pietro, Tondi, Fortini 2024]

  *Eriophorum latifolium* community [Aita, Martini & Orsino 1979]

  *Eriophorum latifolium* community [Ciaschetti, Praleskouskaya, Venanzoni 2024]

  *Eriophorum latifolium* community [Raffaelli, Mori Secci, Mariotti Lippi, Fiorini 1997]

  *Junco articulatae-Caricetum frigidae* Pedrotti 1982

  *Menyanthetum trifoliatae* Steffen 1931

  *Senecio alpinus* and *Deschampsia cespitosa* community [Catorci, Balleli, Gatti, Vitanzi 2008]

  *Tofieldio-Schoenetum* Br. Bl. 1971

  *Veronica beccabunga* community [Gerdol & Tomaselli 1987]

 CARICETALIA FUSCAE Koch 1926

  CARICION FUSCAE Koch 1926 nom. conserv. propos.

  *Carex canescens* community [Ciaschetti, Praleskouskaya, Venanzoni 2024]

  *Carex curta* ande *Spagnum subsecundum* community [Foggi, Gennai, Gervasoni, Ferretti, Viciani, Venturi 2007]

  *Carex frigida* community [Foggi, Gennai, Gervasoni, Ferretti, Viciani, Venturi 2007]

  *Carex nigra* subsp. *nigra* community [Ciaschetti, Praleskouskaya, Venanzoni 2024]

  *Carex rostrata* community [Raffaelli, Mori Secci, Mariotti Lippi, Fiorini 1997]

  *Caricetum echinatae* Gargano, Passalacqua & Bernardo 2007

  *Caricetum echinatae* Gargano, Passalacqua & Bernardo 2007 *Genista anglica* variant

  *Caricetum echinatae* Gargano, Passalacqua & Bernardo 2007 *sphagnetosum fallacis*

  *Caricetum nigrae* Br. Bl. 1915

  *Caricetum nigrae* Br. Bl. 1915 *Carex curta* variant [Foggi, Gennai, Gervasoni, Ferretti, Viciani, Venturi 2007]

  *Caricetum nigrae* Br. Bl. 1915 *Climacium dendroides* variant [Gerdol & Tomaselli 1993]

  *Caricetum nigrae* Br. Bl. 1915 *Drosera rotundifolia* variant [Gerdol & Tomaselli 1993]

  *Caricetum nigrae* Br. Bl. 1915 *Sphagnum girgensohnii* variant [Gerdol & Tomaselli 1993]

  *Caricetum nigrae* Br. Bl. 1915 *Sphagnum subsecundum* variant [Gerdol & Tomaselli 1993]

  *Carici canescentis-Agrostietum caninae* R. Tiixen 1937

  *Drepanocladus exannulatus* and *Juncus filiformis* community [Gerdol & Tomaselli 1993]

  *Drepanocladus uncinatus* and *Juncus filiformis* community [Raffaelli, Mori Secci, Mariotti Lippi, Fiorini 1997]

  *Eriophoretum scheuchzeri* Fries 1913

  *Eriophorum latifolium* community [Foggi, Gennai, Gervasoni, Ferretti, Viciani, Venturi 2007]

  *Eriophorum scheuchzeri* community [Foggi, Gennai, Gervasoni, Ferretti, Viciani, Venturi 2007]

  *Juncus filiformis* ande *Drepanocladus exannulatus* community [Foggi, Gennai, Gervasoni, Ferretti, Viciani, Venturi 2007]

  *Menyanthes trifoliata* community [Raffaelli, Mori Secci, Mariotti Lippi, Fiorini 1997]

  *Sphagno auriculati-Caricetum echinatae* Raimondo & Di Gristina 2021

  *Sphagno inundati-Caricetum stellulatae* Brullo, Scelsi & Spampinato 2001

  *Sphagno-Caricetum nigrae* Gerdol & Tomaselli 1993

  *Sphagno-Caricetum nigrae* Gerdol & Tomaselli 1993 *sphagnetosum compacti* Gerdol &

  Tomaselli 1993

  *Sphagno-Caricetum nigrae* Gerdol & Tomaselli 1993 *Sphagnum russowii* variant

  *Sphagnum flexuosum* community [Raffaelli, Mori Secci, Mariotti Lippi, Fiorini 1997]

  *Trichophorum alpinum* and *Carex davalliana* community [Foggi, Gennai, Gervasoni, Ferretti, Viciani, Venturi 2007]

  *Tricophorum cespitosum* community [Aita, Martini & Orsino 1979]

 SCHEUCHZERIETALIA PALUSTRIS Nordhagen ex Tx. 1937

  CARICION LASIOCARPAE Van Den Bergen 1949

  *Caricetum rostratae* Rubel 1911

  *Menyanthes trifoliata* community [Foggi, Gennai, Gervasoni, Ferretti, Viciani, Venturi 2007]

  SCHEUCHZERION PALUSTRIS Nordhagen ex Tx. 1937

  *Caricetum limosae* Gerdol & Tomaselli 1993

  *Sphagnum flexuosum* community [Gerdol & Tomaselli 1993]
